# Comparison of the Pentax Airwayscope, Glidescope Video Laryngoscope, and Macintosh Laryngoscope During Chest Compression According to Bed Height

**DOI:** 10.1097/MD.0000000000002631

**Published:** 2016-02-08

**Authors:** Wonhee Kim, Yoonje Lee, Changsun Kim, Tae Ho Lim, Jaehoon Oh, Hyunggoo Kang, Sanghyun Lee

**Affiliations:** From the Department of Emergency Medicine, Kangnam Sacred Heart Hospital, Hallym University, Republic of Korea (WK); Department of Emergency Medicine, Guri Hospital, Hanyang University, Republic of Korea (YL, CK); and Department of Emergency Medicine, Seoul Hospital, Hanyang University, Republic of Korea (THL, JO, HK, SL).

## Abstract

We aimed to investigate whether bed height affects intubation performance in the setting of cardiopulmonary resuscitation and which type of laryngoscope shows the best performance at each bed height.

A randomized crossover manikin study was conducted. Twenty-one participants were enrolled, and they were randomly allocated to 2 groups: group A (n = 10) and group B (n = 11). The participants underwent emergency endotracheal intubation (ETI) using the Airwayscope (AWS), Glidescope video laryngoscope, and Macintosh laryngoscope in random order while chest compression was performed. Each ETI was conducted at 2 levels of bed height (minimum bed height: 68.9 cm and maximum bed height: 101.3 cm). The primary outcomes were the time to intubation (TTI) and the success rate of ETI. The *P* value for statistical significance was set at 0.05 and 0.017 in post-hoc test.

The success rate of ETI was always 100% regardless of the type of laryngoscope or the bed height. TTI was not significantly different between the 2 bed heights regardless of the type of laryngoscope (all *P* > 0.05). The time for AWS was the shortest among the 3 laryngoscopes at both bed heights (13.7 ± 3.6 at the minimum bed height and 13.4 ± 4.7 at the maximum bed height) (all *P* < 0.017). The TTI of Glidescope video laryngoscope was not significantly shorter than that of Macintosh laryngoscope at the minimum height (17.6 ± 4.0 vs 19.6 ± 4.8; *P* = 0.02).

The bed height, whether adjusted to the minimum or maximum setting, did not affect intubation performance. In addition, regardless of the bed height, the intubation time with the video laryngoscopes, especially AWS, was significantly shorter than that with the direct laryngoscope during chest compression.

## INTRODUCTION

It is important to perform high-quality cardiopulmonary resuscitation (CPR) in patients with cardiac arrest.^1,2^ Although chest compression (CC) is performed before other procedures during CPR, proper airway management should also be considered immediately in such patients, especially those whose lungs are collapsed from hypoxia, such as from asphyxia, massive oral bleeding, or vomitus.^3^ Emergency endotracheal intubation (ETI) should be conducted to ameliorate hypoxia during CC performance.

The optimal bed height differs between CC and ETI. The optimal bed height for CC is approximately the knee height of the CPR provider.^4,5^ In contrast, to obtain the optimal mechanical advantage, the patient's head should be elevated to the level of the lower part of the intubator's sternum during ETI.^6^ Thus, clinicians may face difficulties when determining the appropriate bed height during CPR.

If the bed height is elevated to its maximum, the patient's head is usually placed at the level of the lower part of the intubator's sternum, which facilitates good intubation performance. However, the CPR provider should use a step stool or kneel on a narrow bed to keep the bed height optimal for CPR, which could make the CPR provider's posture unstable and lower the quality of CC. Conversely, when the bed height is adjusted to knee height for optimal performance of the CPR provider, the intubation performance may be affected.

To the best of our knowledge, no study has investigated the relationship between bed height and successful intubation according to the type of laryngoscope used during CC. Thus, we investigated whether bed height affects intubation performance (intubation time and success rate) in the setting of CPR and which type of laryngoscope shows the best performance at each bed height.

## METHODS

### Study Design

We conducted a randomized crossover manikin study to examine intubation performance using 3 types of laryngoscopes and 2 bed heights during CPR. This study was performed at Hanyang University's simulation center in March 2014. The local ethics committee approved this study in January 2014 (2013–12–009–001). We registered the study protocol in Clinical Trials before study initiation (Clinicaltrials.gov: NCT02074098).

### Equipment and Material

Participants performed ETI using 1 direct laryngoscope and 2 video laryngoscopes. They used endotracheal tubing with an internal diameter of 7.5 mm (Portex, St Paul, MN) and the manufacturer's stylet for intubation. To perform direct laryngoscopy, the Macintosh laryngoscope (MCL) was used; this device has a size-4 curved blade with a Satin Slip Stylet (Mallinckrodt Medical, St Louis, MO). Two types of video laryngoscopes—a Glidescope video laryngoscope (GVL; Verathon Inc., Bothell, WA) and a Pentax-Airwayscope (AWS; Pentax corporation, Tokyo, Japan)—were used in this study. The GVL has a hyperangulated, nonchanneled, standard-size blade and its own rigid stylet, the GlideRite, for intubation. The AWS includes a channeled standard blade.

We used a high-fidelity manikin (SimMan, Laerdal, Stavanger, Norway) to perform CC and ETI. The normal (nondifficult) airway setting was maintained in the manikin during the study. To obtain audiovisual feedback for the depth and rate of CC, Q-CPR (Philips Medical, Andover, MA) was applied during CC.

Two different bed-height settings were simulated using a bed (Transport stretcher No. 747, 76 × 211 cm, 228 kg; Stryker Co., Kalamazoo, MI) with a foam mattress (66 × 192 × 7.6 cm, soft foam with polyurethane covering; Stryker Co., Kalamazoo, MI). A backboard (45 × 60 × 1 cm; 3 kg Lifeline Plastic, Sung Shim Medical Co., Bucheon, Korea) was placed on the bed. The height of the stretcher bed was adjustable between a minimum of 55 cm and a maximum of 91 cm; thus, the minimum bed height was set at 63.6 cm (bed height: 55 cm + foam mattress: 7.6 cm + backboard: 1 cm), and the maximum bed height was set at 99.6 cm. The minimum bed height was appropriate for CC, and the maximum was appropriate for ETI.

The CPR providers used a step stool (39.5 × 45 × 41 cm; Gunica Co., Gyeongsangnam-do, Korea) when they performed CC at the maximum bed height.

### Participants

The sample size was calculated based on a previous study regarding the time required for intubation with CC.^7^ The intubation times (mean ± SD) were as follows: MCL (26.2 ± 9.9 s) and AWS (19.0 ± 7.4 s).^7^ To detect a 33% difference in intubation time with a power of 0.8, we estimated that 15 operators would be adequate for each device with a 20% dropout rate. We recruited physicians working at 1 tertiary medical center in March 2014. We included healthy volunteers who were between 18 and 60 years old and had more than 50 experiences of intubation using MCL. We excluded individuals with wrist or lower back disease. All participants signed a written consent form before being included.

### Interventions

**FIGURE 1:**
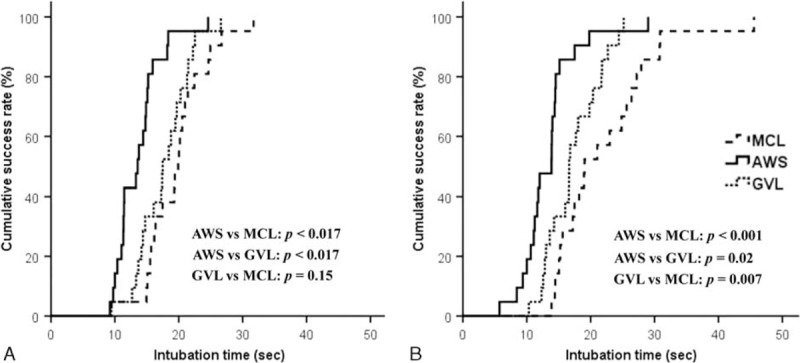
Flow diagram. AWS = Airwayscope; GVL = Glidescope video laryngoscope; MCL = Macintosh laryngoscope.

**FIGURE 2:**
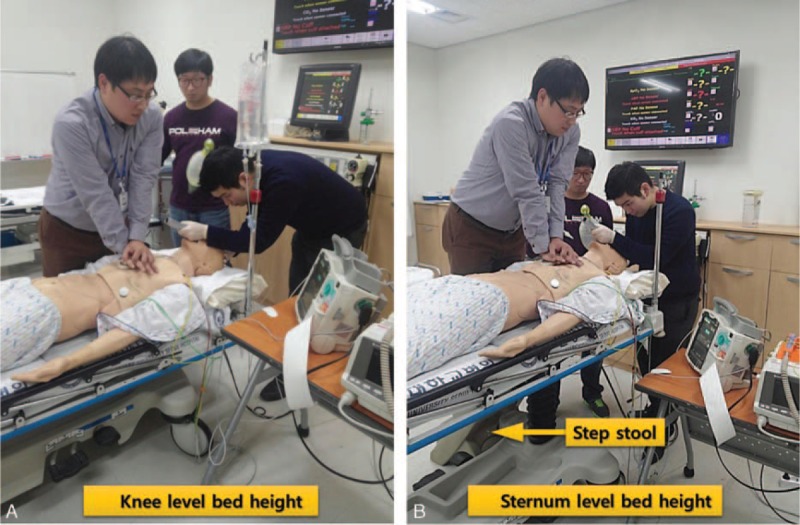
Comparison of cumulative success rate related to total intubation time at (A) knee-level and (B) sternum-level bed heights. A *P* value less than 0.016 was considered statistically significant. AWS = Airwayscope; GVL = Glidescope video laryngoscope; MCL = Macintosh laryngoscope.

All participants completed a brief questionnaire consisting of demographic information (age, sex, body weight, and height) and prior clinical intubation experience with MCL, GVL, and AWS (Table 1). Before starting the trials, the participants had a 10-minute practice period to familiarize themselves with all of the laryngoscopes and perform ETIs on the Laerdal Airway Management Trainer (Laerdal, Stavanger, Norway). Twenty-one participants were enrolled, and they were randomly allocated into 2 groups: group A (n = 10) and group B (n = 11) (Figure 1). After group allocation, the participants performed ETIs in a random order generated by a sequence generator (www.random.org) to minimize the learning effects for intubation. Only 1 attempt at intubation was allowed with each type of laryngoscope. In phase 1, each participant in group A sequentially attempted ETI using each of the 3 types of laryngoscopes according to a given order at the maximum bed height (ETI level) while CC was conducted simultaneously. Each intubation was performed at an interval of 10 minutes (Figure 2). Similarly, each participant in group B (n = 11) attempted the intubations at the minimum bed height (CC level) in the same manner. After a mandatory rest period of 30 minutes, the participants in each group performed intubations at the bed height adjusted to the opposite level (phase 2). The manikin's head and neck were placed in a sniffing position using rolled sheets for MCL. Continuous CC was performed by 2 CPR providers, who alternated for each intubation attempt. These providers were also encouraged to maintain a CC depth of more than 5 cm and a CC rate of more than 100 beats/min using Q-CPR. All of the CPR providers were basic life support (BLS) healthcare providers certified by the American Heart Association (AHA).

**TABLE 1:**
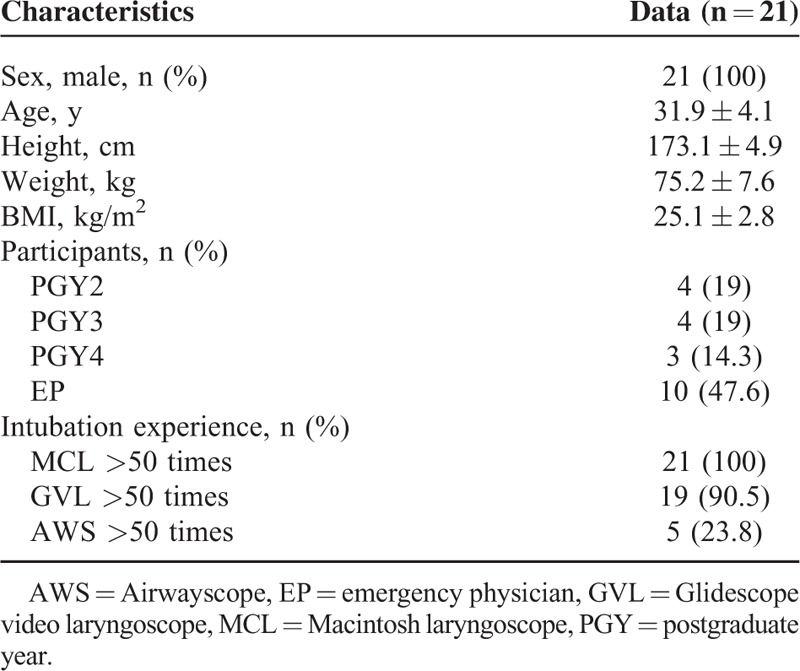
Baseline Characteristics

### Outcomes

The primary outcomes were the time to intubation (TTI) and the success rate of ETI. TTI was defined as the time taken from insertion of the blade into the manikin's mouth to the first ventilation with the Bag-Valve-Mask (BVM). TTI was divided into 2 steps: time to visualization of the glottis (TTV), which was defined as the time from blade insertion into the mouth to the time point of visualization of the glottis; and the time to endotracheal tube progression (TTP), which was measured from the point of visualization of the glottis to the endpoint. Successful ETI was defined as a visible chest rise by bagging with the BVM. Intubation failure was defined as follows: esophageal intubation or exceeding the time limit of 120 seconds.

The glottis view was measured according to the Cormack and Lehane (CL) grade.

### Statistical Analysis

The data were compiled using a standard spreadsheet application (Excel, Microsoft, Redmond, WA) and analyzed using the Statistical Package for the Social Sciences (SPSS) 20.0 KO for Windows (SPSS Inc., Chicago, IL). We generated descriptive statistics and presented them as frequencies and percentages for categorical data and mean with standard deviation (mean ± SD) for continuous data. To compare intubation time among the 3 laryngoscopes, the Friedman test (nonparametric data) or repeated-measures analysis of variance (ANOVA) (parametric data) was used for continuous variables. A post-hoc analysis was conducted with the Wilcoxon rank-sum test (nonparametric data) or paired *t* test (parametric data) using the Bonferroni correction. McNemar test was used to compare categorical variables, such as the success rate for intubation and glottis view. Kaplan–Meier analysis was performed to analyze the cumulative success rate regarding intubation time. A post-hoc analysis was also performed using the log-rank test with the Bonferroni correction. Additionally, multivariate linear regression analysis was conducted to analyze the factors influencing intubation time. For all analyzed data, *P* < 0.05 was considered statistically significant. In contrast, in post-hoc analysis, *P* < 0.017 was considered significant.

## RESULTS

### Baseline Characteristics

Twenty-one participants were enrolled in this study, and none were excluded. The baseline characteristics of the participants are shown in Table 1.

### Intubation Performance at 2 Different Bed Heights

The intubation time and success rate were not significantly different between the 2 different bed heights regardless of the type of laryngoscope (Table 2). In terms of TTI, the time for AWS was the shortest among the 3 laryngoscopes at both bed heights (13.7 ± 3.6 at the minimum bed height and 13.4 ± 4.7 at the maximum bed height) (all *P* < 0.017). The TTI for MCL was longest at both bed heights. The TTI of GVL was significantly shorter than that of MCL at the maximum bed height (17.2 ± 4.2 vs 22.0 ± 7.7; *P* = 0.005), although the difference between these devices was not significant at the minimum height (17.6 ± 4.0 vs 19.6 ± 4.8; *P* = 0.02) (Table 3).

**TABLE 2:**
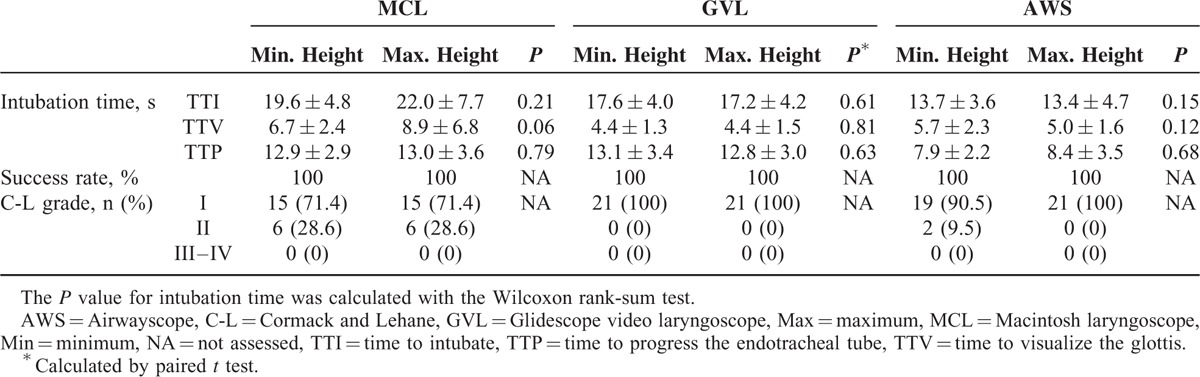
Comparison of Intubation Performance Between 2 Bed Height Settings

**TABLE 3:**
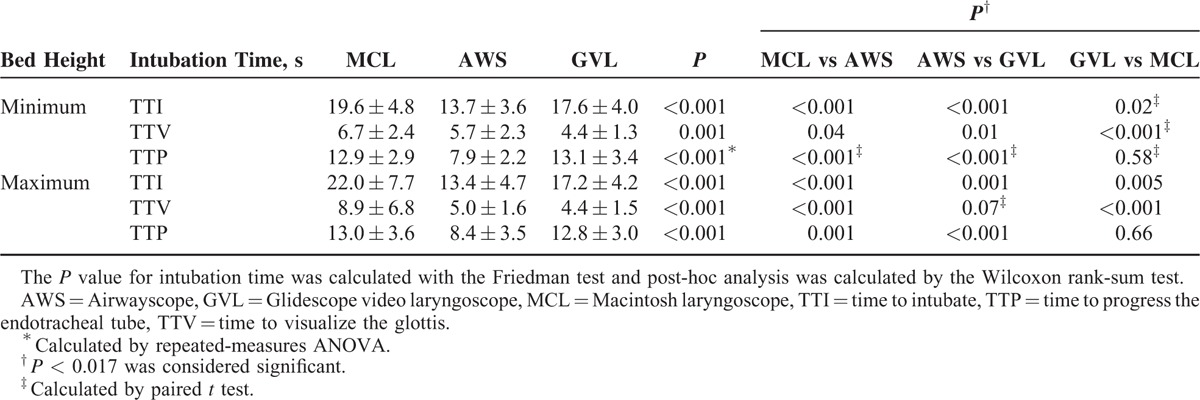
Comparison of Intubation Performance Using 3 Laryngoscopes at Each Bed Height

The TTV of GVL was significantly shorter than that of AWS or MCL at the minimum height (GVL vs AWS: *P* = 0.01; GVL vs MCL: *P* < 0.001). At the maximum height, the TTV of GVL was also shortest among the 3 laryngoscopes, although those of GVL and AWS were not significantly different (*P* = 0.07). For the comparison of TTP, the use of AWS significantly reduced TTP compared with GVL and MCL at both bed height settings (all *P* < 0.017).

The overall success rate of intubation was 100%, and the glottis view was good (CL I–II) regardless of the type of laryngoscope or the bed height level (Table 2).

### Cumulative Success Rate at 2 Bed Heights

**FIGURE 3:**
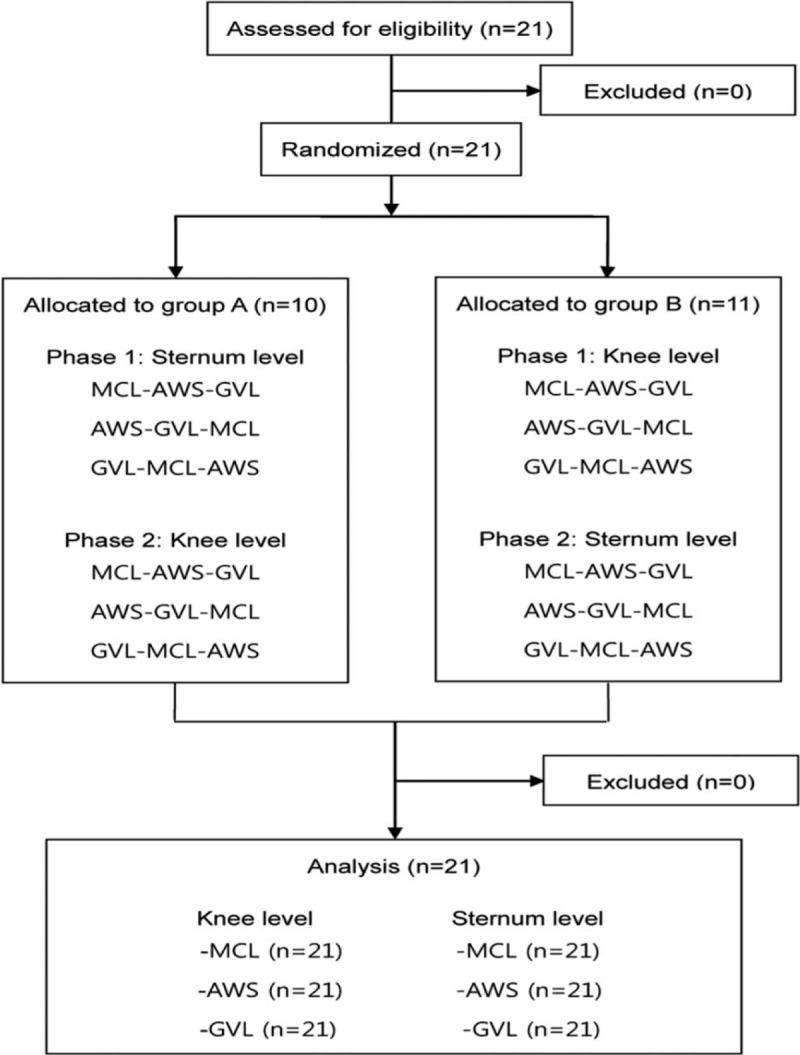
(A) Knee-level and (B) sternum-level bed heights.

Regarding the cumulative success rates, AWS was the fastest among the 3 laryngoscopes at the minimum bed height (all *P* < 0.017); GVL was not significantly different from MCL (*P* = 0.15) at that height. At the maximum bed height, AWS and GVL were faster than MCL (AWS vs MCL: *P* < 0.001; GVL vs MCL: *P* = 0.007). Although AWS was also the fastest at the maximum height, the difference between AWS and GVL was not significant (*P* = 0.02) (Figure 3).

### Factors Influencing Intubation Time

The intubation time (time to complete) was affected by whether the participants had passed their emergency medicine boards, the participants’ intubation experience, and the type of laryngoscope. In contrast, the bed height and the experience with GVL were not independent factors affecting intubation time. Emergency physicians could perform intubation faster than residents (*P* < 0.001). Additionally, significant experience with AWS facilitated shortening the intubation time (*P* = 0.03). The use of either of the 2 video laryngoscopes also shortened the intubation times compared with MCL (AWS, *P* < 0.001; GVL, *P* < 0.001) (Table 3).

## DISCUSSION

At the optimal bed height for ETI, the lower part of the intubator's sternum is placed at the patient's head.^6^ Given that the maximum bed height of a conventional stretcher is approximately 100 cm, which is near the intubator's waist, the bed must be elevated to meet the optimal bed height for ETI. However, at that maximal bed height, the chest compressor should use a step stool or kneel on the bed to be at the optimal bed height for CC. If there is no immediately available step stool, high-quality CC cannot be provided at that bed height. Additionally, the bed in the resuscitation room is generally narrow; thus, kneeling on such a narrow bed could decrease the CC quality. Therefore, in the case of CPR, the bed height may need to be immediately adjusted to knee level to maintain high-quality CC. Roberts et al^6^ demonstrated that the intubator must keep his or her back straight and should not hunch over the patient. Thus, we originally thought that physicians might have difficulty intubating at such a low bed height because bending so low might disturb their ability to maintain the glottis view and handle the laryngoscope. However, de Laveaga et al^10^ reported that the intubation time using MCL was not decreased when the bed height was adjusted to a height of 62 cm (approximately knee level) compared with a height of 96 cm. However, the influence of CC and the use of a video laryngoscope were not considered in that study. In this study, even during CC, the intubation performance (intubation time and success rate) did not decrease, regardless of the type of laryngoscope, when the bed height was adjusted to knee level (minimum height), which is the appropriate height for CC. At that height, the use of AWS was able to significantly reduce the intubation time compared with GVL and MCL. Thus, the bed height should be selected to achieve high-quality CCs rather than to suit the intubator during CPR.

In previous studies, the video laryngoscope has shown better intubation performance than direct laryngoscopes during CC at one bed height of 80 to 100 cm. Additionally, AWS has been shown to be superior to MCL at decreasing both TTV and TTP, whereas GVL has been shown to be superior to MCL at reducing TTV only.^8,9,13,14^ We also found that compared with MCL, the use of AWS significantly decreased both TTV and TTP, thereby reducing TTI at both bed heights. GVL also significantly reduced TTV, but it did not reduce TTP compared with MCL (the TTP of GVL was longer than that of MCL at the minimum bed height). As a result, the use of GVL only slightly shortened TTI at the minimum bed height, although the TTI of GVL was significantly shorter than that of MCL at the maximum bed height. This result implies that both AWS and GVL are superior to MCL for decreasing the TTV because of the attached camera; however, only AWS is superior to MCL at reducing the TTP. We believe that the prechanneled blade and preequipped endotracheal tube of AWS are likely to be the main factors contributing to decreasing the TTP, although the absence of a stylet in AWS could also be a factor.^11,12^ Thus, a video laryngoscope with a prechanneled blade, such as AWS, is the best option for rapidly viewing the glottis and achieving progression during CPR.

The significant factors affecting the intubation time during CPR were intubation experience and the type of laryngoscope, not the bed height (Table 4). In our previous study, the level of training (intubation experience) of the emergency medicine resident was also an independent factor affecting the success rate of the first ETI attempt in emergency departments.^15^ Han et al^12^ reported that less experienced physicians showed significantly lower intubation success rates with MCL compared with video laryngoscopy when CC was performed even under normal airway conditions. In contrast, Shin et al^16^ demonstrated that experienced physicians could effectively perform ETI with MCL regardless of accompanying CC under normal airway conditions. Therefore, experienced physicians can perform intubation in any situation.

**TABLE 4:**
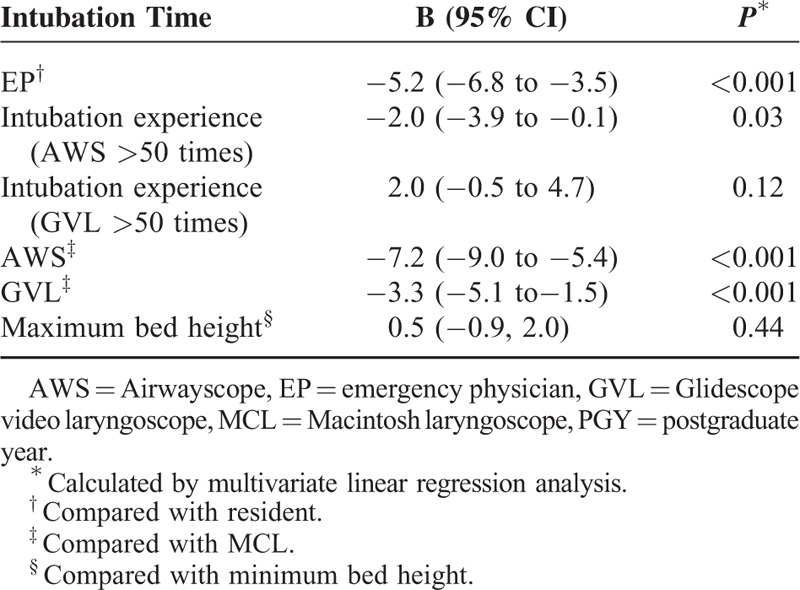
Factors Influencing Intubation Time

There were several limitations to this study. First, we used a high-fidelity manikin reflecting only a normal airway setting during CPR. However, cardiac arrest patients may exhibit difficult airway conditions, which could significantly interfere with emergent intubation. Hence, the measured intubation performance in this study could differ from that of actual CPR. Second, we did not consider the impact of the increasing number of tasks involved in CPR. During CPR, multiple tasks must be performed at the same time, including defibrillation, preparation for intubation, intravenous drug administration, and others. Performing these tasks simultaneously could influence the actual intubation time. Third, the participants included in this study as intubators were all young men. Women or the older population could exhibit different intubation performances compared with the current participants. Furthermore, because relatively experienced intubators with more than 50 attempts at intubation were included in this study, less experienced intubators could result in different findings.

In conclusion, adjusting the bed height to the minimal or maximal level did not affect intubation performance. In addition, regardless of the bed height, the intubation time with video laryngoscopes, especially AWS, was significantly shorter than that with direct laryngoscopes during CC. Therefore, we can conclude that when the emergency intubator reduces the bed height to the minimum level to achieve high-quality CCs, AWS might be the best option to use during CPR.
